# Soil Amendments and Foliar Melatonin Reduced Pb Uptake, and Oxidative Stress, and Improved Spinach Quality in Pb-Contaminated Soil

**DOI:** 10.3390/plants12091829

**Published:** 2023-04-29

**Authors:** Naeem Iqbal, Hafiz Syed Tanzeem-ul-Haq, Gull-e-Faran, Veysel Turan, Muhammad Iqbal

**Affiliations:** 1 Department of Environmental Sciences and Engineering, Government College University, Faisalabad 38000, Pakistan; 2 Department of Biochemistry, Institute of Biochemistry, Biotechnology and Bioinformatics, The Islamia University of Bahawalpur, Bahawalpur 63100, Pakistan; 3 Department of Soil Science and Plant Nutrition, Faculty of Agriculture, Bingöl University, 12000 Bingöl, Turkey; vturan@bingol.edu.tr

**Keywords:** melatonin, biochar, Pb-polluted soil, oxidative stress, microbial numbers, rhizosphere

## Abstract

Amending Pb-affected soil with biochar (BH) and magnesium potassium phosphate cement (MKC) reduces Pb uptake in plants. Moreover, foliar applications of melatonin and proline are also known to reduce plant oxidative stress and Pb uptake. However, little is known about combining both techniques, i.e., adding a combo immobilizing dose (CIA = mixture of BH and MKC at 50:50 ratio) in Pb-polluted soil and foliar application of proline and melatonin for reducing Pb uptake and oxidative stress in spinach. Control, proline, melatonin, CIA, CIA+proline, and CIA+melatonin were the treatments utilized in this pot study to see their effects on reducing plant oxidative stress, Pb uptake, and improving spinach quality in Pb-polluted soil. Moreover, Pb bioavailability, enzymatic activities, and numbers of bacteria, fungi, and actinomycetes in the soil were also evaluated. The effect of CIA on reducing Pb in the soil-plant system and improving soil enzymes and microbial numbers was more pronounced than melatonin alone. The most effective treatment was CIA+melatonin reducing Pb availability in soil (77%), shoots (95%), and roots (84%), alleviating oxidative stress, and improving plant biomass (98%) and nutrients. Soil enzymatic activities and the number of microorganisms in the rhizosphere were also highest with CIA+melatonin. Results highlight the significance of CIA+melatonin, as an inexpensive approach, in remediating Pb-polluted soil and improving spinach quality. However, further research is needed to understand the significance of CIA+melatonin on different crops and various soil Pb concentrations before employing this technique commercially in agriculture and environment sectors.

## 1. Introduction

Natural sources such as volcanic eruptions, wildfires, dust storms, and weathering of parent rocks and anthropogenic activities like smelting, vehicle emissions, and untreated effluents from various industries contaminate the soil with Pb [1,2]. In many countries, mining activities, either in the past or ongoing, have polluted the surrounding arable land and groundwater with Pb [3,4]. To ensure the sustainability of mining operations, mining non-energetic minerals must strike a balance between its goals and the goals of other economic interests, as well as with the larger social and environmental interests [5]. In Pakistan, untreated effluents from Pb–acid batteries, paints, tanneries, and alloys industry are the primary sources of Pb contamination in soil [6].

When plants uptake lower concentrations of Pb, phytochelatins (PCs) are biosynthesized in the roots, which form PCs–Pb complexes and thus reduce Pb mobility to the aerial parts [7,8]. However, a rise in the Pb accumulation beyond the threshold level reduces the uptake of water and nutrients, respiration, transcription, photosynthesis, and N assimilation in plants [6,9]. Moreover, reactive oxygen species (ROS) are originated in plants, ruining cell membranes along with cellular macromolecules for instance proteins, lipids, and nucleic acids, giving rise to cellular death [1,10].

The permissible limit for Pb in edible plant tissues is 5 mg kg^−1^ [11]. Leafy vegetables, especially spinach, lettuce, and coriander, tend to accumulate higher concentrations of Pb from the soil than others [12]. Several health issues associated with cardiovascular, nervous, renal, reproductive, and immune systems are reported in humans upon consuming leafy vegetables containing Pb [1,2]. Unfortunately, different leafy vegetables having Pb concentrations exceeding the WHO/FAO [11] permissible limit are present in the local markets of Pakistan [12].

In situ stabilization of Pb in soil with cheap inorganic and organic amendments reduce Pb mobility in soil and plant [13]. Biochar (BH) is a carbon-based material that is prepared by pyrolyzing (above 350 °C) different organic wastes and has attractive physico-chemical characteristics like high moisture retention, porosity, cation exchange capacity (CEC), a large surface area, and various functional groups [10]. However, several factors like feedstock cost, price to activate BH, and the large quantity of BH needed for ecological restoration have limited its application at a larger scale. Alternatively, BH production from the digestate of biogas plants is far cheaper than other feedstocks. Production of BH from digestate can reduce the production cost of BH and expand its commercial applicability [14]. Adding BH to the soil reduces Pb uptake in plants by reducing soil Pb mobility through rising soil pH and binding Pb on large surface areas and functional groups through ion exchange, precipitation, and surface complexation [15]. Magnesium potassium phosphate cement (MKC), a cheap cementitious material, stabilizes Pb by converting it into insoluble compounds like Pb-phosphate [Pb_3_(PO_4_)_2_], pyromorphite [Pb_5_(PO_4_)_3_X, X = Cl^−^, OH^−^, F^−^] and struvite-K encapsulation, and through raising soil pH [16]. Incorporating MKC in Pb-polluted soil efficiently lowered Pb concentrations in pea grain (53%) and plant-accessible Pb fraction in soil (42%) than unamended soil. Interestingly, Pb concentrations in spinach leaves and DTPA extract were reduced by 85% and 73%, respectively, after conditioning Pb-polluted soil with a combo immobilizing amendment (CIA), prepared from BH and MKC (50:50 ratio) [6].

Under Pb stress, plant cells produce proline, a proteinogenic amino acid that reduces Pb stress by chelating Pb ions [17]. The application of proline to the plants protected the photosynthetic apparatus and enhanced the physiology, water relations, antioxidant defense, biochemistry, and nutrition of a number of plant species growing on metal-contaminated soils [18,19,20]. Similarly, melatonin (*N-acetyl-5-methoxytryptamine*) is a bioactive molecule produced in plant cells subjected to diverse stresses, such as Pb stress. The exogenous application of melatonin reduces Pb translocation to aerial plant parts by chelating Pb with PCs and then compartmentalizing PCs–Pb complexes within the roots [21]. Melatonin spray enhances the ability of the plant to overcome oxidative stress [17]. It improved the morphological and physiological attributes of maize [21] and safflower [17] grown under Pb stress.

We hypothesize that adding CIA in Pb-polluted soil and the foliar application of melatonin or proline may have additive effects to minimize Pb movement in soil and plants. This cost-effective technique can reduce Pb pollution in soil and minimize contamination of food, surrounding land, and groundwater from Pb. Therefore, we performed a pot study using Pb-polluted soil having soil addition of CIA, foliar application of melatonin and proline, as sole treatments, and combining CIA with melatonin and proline. The study aimed to see treatment effects on plant growth, nutritional value, oxidative stress, and Pb transfer in plants.

## 2. Materials and Methods

### 2.1. Experimental Soil

The soil was taken from “Lalazar plant supplies” in Faisalabad, Pakistan. Soil was shade dried and passed across a stainless steel netting (2 mm) to clear it from various artifacts. To quantify different characteristics of this soil, a sub-sample was analyzed by adopting different standard laboratory procedures properly described in our previous work [6,20] and are presented herewith (Table 1).

A specific quantity (72 kg) of experimental soil was considered for spiking with Pb (600 mg kg^−1^ soil) utilizing Pb(NO_3_)_2_;. Next, the spiked soil was thrice autoclaved (121 °C for 2 h, with 24 h intervals between autoclaving events) to kill indigenous microorganisms. Later, the soil was moistened (65% of the water holding capacity (WHC)) with sterile de-ionized water, sealed in ziplock bags, and placed in a dark room (at 25 °C) for 2 months. During this incubation, each ziplock bag was regularly (with an interval of five days) weighed and opened, the soil was sprayed with a known amount of sterile de-ionized water to maintain 65% WHC, and properly mixed with a sterilized spatula to avoid the growth of bacteria, algae, and fungi. Later, this incubated soil was air-dried and analyzed to quantify the concentrations of bioavailable and total Pb. To assess the soil total Pb concentration (Total(Pb)), a method of Chen and Ma [22] was chosen. The soil samples were digested in aqua regia (a blend of HCl and HNO_3_) having an amount of 3:1) and further assessed on an ICP–MS (PerkinElmer’s NexION^®^ 2000, Waltham, MA, USA). The bioavailable Pb concentration in the soil was estimated through soil extraction with a diethylenetriamine pentaacetate (DTPA) (5 mM) solution (1:2, soil to DTPA ratio). Later, the Pb in extract (PbDTPA) was valued on ICP–MS [23]. Achieved data (mean ± standard deviation, *n* = 3) were as follows: PbDTPA = 3.10 ± 0.02 mg kg^−1^ soil and total (Pb) = 621.2 ± 11.1 mg kg^−1^ soil.

After spiking, we could only extract a very low concentration of PbDTPA from soil. The fate of added Pb into the soil to become less bioavailable is as follows. Once the Pb enters the soil environment, it goes through various physicochemical phases (soluble/exchangeable phase, binding with organic matter, carbonates, oxides of Fe and Mn, and soil mineral matrix), which govern the mobility and bioavailability of Pb [24]. Lead has a great affinity towards soil organic matter (SOM). The carboxylic groups of humus ionize as the pH values increase while forming strong complexes with Pb through ion exchange and complexation [24]. Moreover, the CEC refers to the function of negatively charged sites on soil colloids that attract Pb and form electrostatic bonds with it. Different clay minerals in the soil also strongly adsorb Pb [25]. In addition to it, several other mechanisms are also involved that reduce the bioavailability of Pb in the soil, which are as follows: (a) the oxides of Fe and Mn strongly adsorb Pb [25], (b) Pb reacts with calcium carbonate (CaCO_3_), which results in the formation of insoluble lead carbonate (PbCO_3_) [26], and (c) the available P reacts with Pb and transforms it into highly insoluble compounds such as Pb-phosphate and pyromorphite [26]. These processes fixed the spiked Pb in the soil and reduced its bioavailability. The plants can only utilize P present in CaP (compound of phosphate with calcium), while to some extent, AlP (compound of phosphate with aluminum). Whereas, FeP (compound of phosphate with iron) and other forms, such as Pb-phosphate and pyromorphite, are not accessible to plants and thus cannot result in the accumulation of Pb in the food chain [6,27].

### 2.2. Foliar Sprays and Soil Amendment

In this study, CIA was formulated by mixing *Bougainvillea alba* L derived BH with MKC by maintaining the 50:50 ratio. A high-quality proline (Pyrrolidine–2–carboxylic acid, L–Proline) was purchased from Sigma-Aldrich, Taufkirchen, Germany. The proline solution (20 mM L^−1^) was prepared by dissolving the proline in distilled water containing a 0.1% surfactant named “Tween-20”. Likewise, the melatonin was gained from Sigma-Aldrich (St. Louis, MI, USA). To apply on foliage, a melatonin solution (10 mM L^−1^) was prepared by dissolving melatonin in 100% ethanol and stored (at −20 °C). This stored melatonin solution was further diluted at the spray time until the necessary concentration of 100 µM L^−1^. Previously, different doses of melatonin [17,21] and proline [20] were able to reduce the concentrations of Pb and other heavy metals in different parts of the plants. Based on the recommendations of these studies, the most effective doses of proline (20 mM L^−1^) and melatonin (100 µM L^−1^) were selected.

### 2.3. Experimental Setup

The Pb-contaminated soil was used to prepare six treatments named control, proline, melatonin, CIA, CIA+proline, and CIA+melatonin (Table 2). To prepare the soils for CIA, CIA+proline, and CIA+melatonin treatments, the CIA was distinctly mixed (3% *w*/*w* of the soil) with three separate masses of Pb-contaminated soil (each weighing up to 15 kg) in three plastic buckets using different spatulas. Next, these three soil mixtures were separately homogenized in a mechanical shaker. Since the CIA contained both BH and MKC (at a 50:50 ratio), amending the soils of CIA, CIA+proline, and CIA+melatonin treatments with CIA (3% *w*/*w* of soil) provided 15 g BH kg^−1^ soil (1.5% *w*/*w* of soil) and 15 g MKC kg^−1^ soil (1.5% *w*/*w* of soil). Three separate masses of Pb-contaminated soil (each weighing up to 15 kg) were kept to formulate three other treatments named control, proline, and melatonin. The melatonin and proline treatments did not receive soil amendment (CIA), but melatonin and proline spray on the plants. The control treatment neither received soil amendment (CIA) nor any foliar spray (melatonin or proline) on the plants.

These treated soils were separately moisturized (65% WHC) and stored (25 °C) in a dark location for 14 days. After every five days, the treated soils were sprayed with a known amount of de-ionized water to maintain 65% WHC and properly mixed with the help of sterilized spatulas to avoid microbial growth. Next to incubation, the polyvinyl chloride (PVC) pots (width across = 24.8 cm and height = 34 cm) having drain holes underneath were attentively filled with 5 kg of treated and untreated soils by following the experimental layout (Table 2). Each treatment was repeated three times. Coming forth, these pots were transported to the experimental site (Government College University Faisalabad, Pakistan) and arrayed in a completely randomized design (CRD). The experimental site has excellent ambient status (temperature ≈ 30 °C, illumination = 7.8–9.7 h, and moisture ≈ 35%) for plants to grow. Spinach seeds (sort, Red Cardinal) were dispersed in an individual pot and covered with a thin layer of coco peat. The plants emerged after four days. Successively, three healthy plants per pot were maintained. A recommended dose (1 g kg^−1^ soil) of fertilizer named “Grow Fertilizer 18-18-18” was once applied to each pot after one week of plant germination. The nutrient composition of this fertilizer was as follows: N = 18%, P = 18%, K = 18%, S = 2%, B = 0.02%, Cu = 0.05%, Fe = 0.13%, Mn = 0.07%, and Zn = 0.05%. After fifteen days of plant emergence, they were foliar sprayed with melatonin (100 μmol L^−1^) and proline (20 mM L^−1^) with the help of manual spraying equipment in the evening till the leaves were dipping. Moreover, the plants growing in the treatment pots where no sprays of melatonin and proline were planned received a spray of an equivalent volume of double-distilled water. During the entire period of this experiment, the plants were irrigated with distilled water while considering the atmospheric situation of moisture. The plants were harvested on the 45th day of growth when they became mature. Before plant hoarding, the plant heights were gauged by a measuring reel.

### 2.4. Experiment Termination

A sharp plant cutter was used to reap the above-ground plant biomass. The roots were also recovered from all pots. Later, the roots were wiped with a nylon brush to retrieve the rhizosphere soil [28]. Next to this, soil (up to 20 g) from the distance (2–5 cm) of the roots and their surroundings was gathered and referred to as bulk soil [29]. Both soil portions were discretely air-dried, sieved (<2 mm), and stored for analysis. Next to the hoarding process, the shoots fresh weight (Shoots FW) and roots fresh weight (Roots FW) were computed. After air-drying, this plant biomass was oven-dried (at 60 °C), and the shoots dry weight (Shoots DW) and roots dry weight (Roots DW) were recorded. The plant biomass was secured for further analysis.

### 2.5. Soil Analysis

#### 2.5.1. Lead Bioavailability in Soil

A sub-sample (5 g) from the ex situ soil was considered to assess the concentration of PbDTPA by following the protocol of [23] via the extraction of soil with DTPA solution (1:2 ratio). Similarly, the PbH_2_O in soil was obtained via a 1:5 soil to de-ionized water suspension. Later, the Pb concentrations in both of these extracts were measured with the help of ICP–MS.

#### 2.5.2. Microbial Numbers and Microbial Biomass Carbon

The standard methodologies of Li et al. [30] and Wu et al. [31] were used for counting microbial numbers in both bulk and rhizosphere soils. To achieve it, aqueous extracts from 3 g of fresh soil samples were serially diluted and spread on potato dextrose agar medium for fungi, beef extract-peptone medium for bacteria, and Gause’s synthetic agar medium for actinomycetes. Next to incubation, the microbial numbers were counted at the appropriate condition for 2–5 days. Likewise, microbial biomass carbon (MBC) in the soil was quantified using the chloroform fumigation protocol [32].

#### 2.5.3. Soil Enzymatic Activities

To measure urease activity, a soil sample (1 g) was mixed with urea solution (0.5 mL) and borate buffer (4 mL, pH = 10.0). First, such formulated mix was incubated (37 °C for 2 h). Then, 6 mL of potassium chloride (KCl) (1 M) was supplemented, and the flasks were allowed to halt for 60 min. Coming, filtrate was combined with sodium dichloroisocyanurate (C_3_Cl_2_N_3_NaO_3_), Na salicylate/sodium hydroxide (NaOH), and water. This reaction mixture was allowed to react (25 °C, 30 min) to identify the ammonium (NH_4_^+^) quantity preceding the determination of the optical density of mixture (690 nm) [33]. Likewise, hydrogen peroxide (H_2_O_2_) used up by soil was observed to estimate catalase activity [34]. To achieve it, a mixture was formulated by adding 25 mL of H_2_O_2_ (3%) to a 5 g soil mass and incubated (4 °C) for 60 min. Succeeding, 25 mL of sulfuric acid (H_2_SO_4_) (1 M) was mixed. The contents were filtered, and 20 mL of H_2_SO_4_ (0.5 M) was supplemented to 5 mL filtrate. Lastly, this solution was titrated against potassium permanganate (KMnO_4_) (5 mM) to estimate non-reactive H_2_O_2_.

Similarly, a procedure defined by study [35] was followed to assess the acid phosphatase activity in the soil. The ρ-nitrophenyl phosphate content was determined in reaction blend, and the absorbance was spectrophotometrically noted from 400 to 420 nm. To estimate phosphomonoesterase activity, the method of Paz-Ferreiro et al. [36] was used. Standard curve was drawn for measuring the phosphomonoesterase. β-glucosidase activity was determined by use of protocol of Eivazi and Tabatabai [37]. For this purpose, the soil (1 g) was mixed with a ρ-nitrophenyl-β-D-glucopyranoside in the presence of an adjusted pH buffer (pH = 9). After incubating the prepared suspension, the tris (0.02 mol L^−1^, pH = 12) was further blended to stop this reaction. Later, substrate cleavage was formed to release ρ-nitrophenol glucoside, which was noticed at 464 nm on spectrophotometer (Analytic Jena Specord 250 Plus, UK). To quantify protease activity, soil sample (200 mg) was mixed with a buffered casein solution (5 mL, pH 8.1) and TRIS buffer (5 mL, 50 mM, pH 8.1). This mixture was incubated at 50 °C for 2 h. Later, the free aromatic amino acids were extracted with the help of trichloroacetic acid (0.92 M) and measured on a colorimeter utilizing a Folin–Ciocalteu reagent [38].

Cellobiohydrolase activity in the soil was assayed using p-nitrophenyl-β-d-cellobioside in microplates [39]. For this purpose, a reaction mixture was prepared, which had 0.16 mL of 1.2 mM p-nitrophenyl-β-d-cellobioside in 50 mM sodium acetate buffer (pH 5.0) and 0.04 mL soil extract. This mixture was incubated for 2 h at 40 °C. Later, the reaction was halted through adding 0.1 mL of 0.5 M sodium carbonate. Absorbance of the mixture was noted on a spectrophotometer (400 nm). For measuring chitinase activity, 100 mg of soil sample was mixed with citrate phosphate buffer (400 µL, pH 5.8) and paranitrophenyl N-acetyl glucosaminide (100 µL, 5 mM). This mixture was incubated at 37 °C (2 h). Later, the chitinase activity was assessed by computing the released para–nitrophenyl (p–NP) from the reaction blend on a spectrophotometer (at 400 nm) [40].

### 2.6. Plant Analysis

#### 2.6.1. Lead Concentrations in Plants and Leaf Nutrients

The milled samples (0.5 g) of roots and shoots were digested in a prepared mixture of HNO_3_ and HClO_4_, having a ratio of 2:1 (*v*/*v*) [41]. Lead concentrations in the digested samples of shoots and roots were determined on ICP–MS. Mg, Ca, Zn, Fe, K, and Mn concentrations in shoot digests were also valued on ICP–MS. Likewise, the contents of P and N in the digest of shoots were quantified using procedures of Chapman and Pratt [42] and Buresh et al. [43], accordingly.

#### 2.6.2. Calculating BAF(ST), BAF(RT), and TF(ST)

The values of BAF(ST), BAF(RT), and TF(ST) were calculated by using the Formulas (1)–(3) [44].

BAF(ST) = ST(Pb)/Total(Pb)(1)

BAF(RT) = RT(Pb)/Total(Pb)(2)

TF(ST) = ST(Pb)/RT(Pb)(3)

The values for ST(Pb) and RT(Pb) were presented as mg kg^−1^ DW (shoots and roots, respectively), whereas soil total Pb concentration in mg kg^−1^ DW soil.

#### 2.6.3. Photosynthetic Pigments and Relative Water Content

A method defined by Hiscox and Israelstam [45] was used to determine the chlorophyll-a (CLR–a) and chlorophyll-b (CLR–b) contents in fresh leaves. 1.0 g leaf fragment was mixed in 20 mL of a mixture of methanol, chloroform, and water (at ratios 12:5:3). Next, the pigment concentrations were measured at 664.5 nm (CLR–a) and 647.4 nm (CLR–b) on a spectrophotometer.

To calculate turgid leaf weight (TW), the fresh leaves were drenched (25 °C) in de-ionized water and sited in a dark place (24 h). Next, desiccation of leaves (at 60 °C for 48 h) was carried out in an oven to obtain DW. Finally, Equation (4), defined by Mullan and Pietragalla [46], was applied to determine relative water content (RTW) of the plants as follows:

RTW (%) = [(FW − DW)/TW − DW)] × 100(4)

#### 2.6.4. Antioxidant Defense System

For quantification of antioxidant enzymes, 500 mg of a fresh leaves was homogenized in 5 mL of potassium phosphate (K−P) extraction buffer (having pH at 7) composed of 1.0 mM ascorbate, 100 mM KCl, glycerol (10% (*w*/*v*)), and 5 mM β-mercaptoethanol. After centrifugation of this homogenate at 11,500× *g* for 10 min, this supernatant was utilized to measure the activities of ascorbate peroxidase (APX), superoxide dismutase (SOD), and catalase (CAT), as well as the contents of ascorbic acid (AsA), and dehydroascorbate reductase (DHAR). Additionally, the contents of malondialdehyde (MDA) and H_2_O_2_, as well as superoxide anion (O_2_^•−^) in leaves, were estimated with diverse methods (Table 3).

#### 2.6.5. Leaf Biochemistry

Spinach leaves were analyzed for calculating the protein, flavonoids, amino acids, fiber, and fat contents through standard techniques. A Bradford colorimetric method was followed to appraise total protein in leaf sample while taking into account bovine serum as a standard [55]. Likewise, the contents of amino acids were quantified using the leaf ethanol extract. The amino acids were estimated after adding ninhydrin into the harvested extract [56]. To measure flavonoids, leaf extract was mixed into potassium acetate (CH_3_COOK) (1 M, 0.1 mL), aluminum chloride (AlCl_3_) (10%, 0.1 mL), methanol (1.5 mL), and de-ionized water (2 mL). Later on, a standard curve was sketched to measure flavonoids (at 417 nm) on a spectrophotometer [57]. The dry extraction methodology outlined by AOAC [58] was used to ascertain the fat content. The extraction tube was filled with the dried sample (1 g) wrapped in filter paper. The sample was next combined with petroleum ether in glass vial before fixing on the Soxhlet device. The ether evaporated throughout the four to six siphoning cycles, transferring the extract to a glass dish. To evaluate the amount of fat in this dish, it was dried in an oven (at 105 °C) and then cooled. Similarly, 1.25% sodium hydroxide (NaOH) and 1.25% sulfuric acid (H_2_SO_4_) were used to digest the fat-free leaf sample. This chemical mixture was then dried in an oven and burned in furnace. Crude fiber was then calculated as per weight lost during cremation, and left over organic residue was referred to as crude fiber [59].

### 2.7. Quality Assurance and Quality Control

The blank samples and certified reference materials (DCI 7004 for soil analysis and CTA–OTL–1 for plants) were used for quality assurance and control. Recoveries of Pb from certified reference materials were 92–96% (soil) and 94–97% (plant). All glassware and other consumables used to analyze plant and soil samples were initially soaked in diluted HNO_3_ (12 h) and later frequently rinsed with de-ionized water. The chemicals and reagents were procured from Sigma-Aldrich (St. Louis, MI, USA) and UNICHEM (H-6760, Kistelek, Tanya 491, Hungary) [20] All of the used chemicals were of analytical grade.

### 2.8. Statistical Analysis

A CRD was considered by including each treatment repeated threefold to perform this study. Means and standard errors (SEs) were calculated from three replicates using Microsoft Excel 2013^®^. First, a normality test was applied to the data to assess whether the data were normally distributed or not. It was found that the data were normally distributed. Next, data interpretation was conducted through one way of analysis of variance (ANOVA) with the statistical software Statistix 8.1 (Analytical Software, Tallahassee, FL, USA). A least significant difference test (LSD) was opted to detect a distinction (*p* < 0.05) between the means of treatments [60]. This significant difference was depicted with lowercase letters. Finally, the data (parameter-wise) were presented in either tables or figures

## 3. Results

### 3.1. Lead Concentrations in Plant Parts and Plant-Accessible Pb

**Figure 1:**
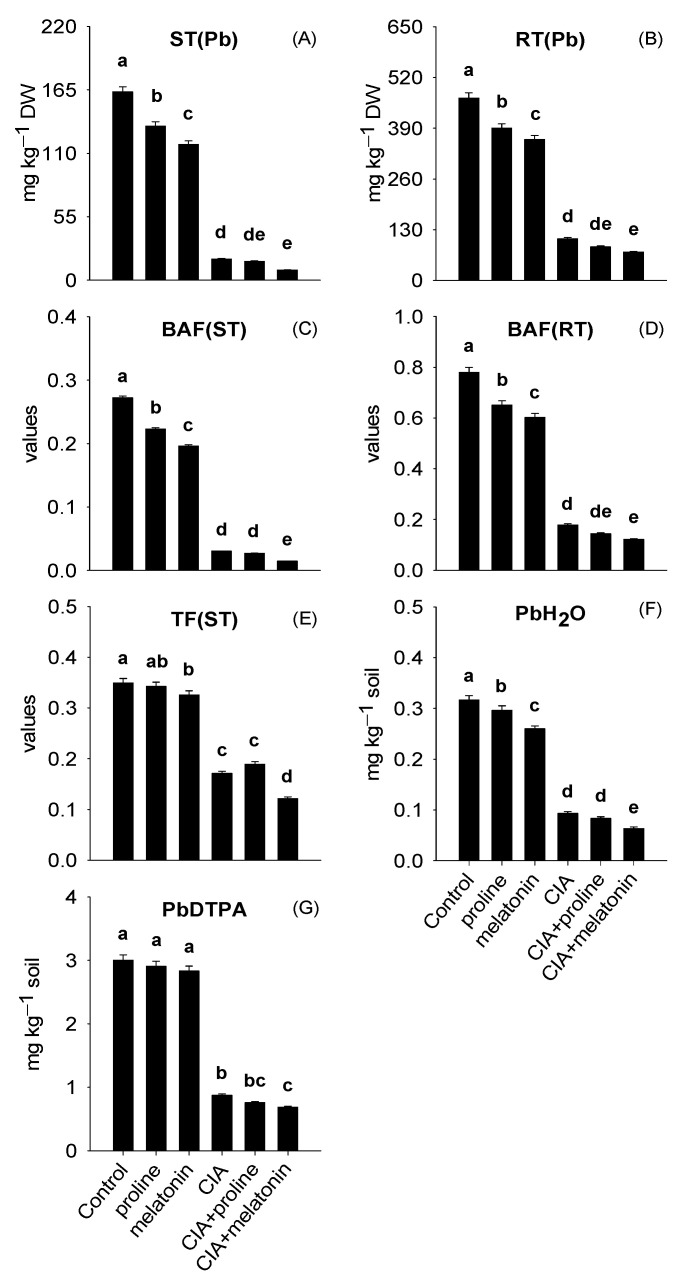
Concentrations of Pb in shoots (ST(Pb)) (**A**), roots (RT(Pb)) (**B**), values of bioaccumulation factor of shoot (BAF(ST)) (**C**), root (BAF(RT)) (**D**), translocation factor of shoot (TF(ST)) (**E**), PbH_2_O (**F**), and PbDTPA (**G**) as affected by conditioning Pb-polluted soil with CIA and foliar application of proline and melatonin, as a single treatment or combining both techniques. Data shown in each bar are mean ± SE (*n* = 3). Significant variations (*p* < 0.05) based on the LSD test are shown by bars with different lowercase letters.

The data of ST(Pb) and RT(Pb) were in the ranges of 8.87–163.3 and 73.0–467.7 mg kg^−1^ DW, respectively (Figure 1A,B). BAF(ST), BAF(RT), and TF(ST) values were in the ranges of 0.02–0.27, 0.12–0.78, and 0.12–0.35 (Figure 1C–E). The PbH_2_O in soil and PbDTPA ranged from 0.059–0.32 and 0.68–3.00 mg kg^−1^ soil (Figure 1F,G). The highest reductions in the ST(Pb) by 95%, RT(Pb) by 84%, and PbDTPA by 77% were because of CIA+melatonin, respectively, against control treatment. Foremost reductions in BAF(ST), BAF(RT), and TF(ST) values, and the PbH_2_O in soil by 95%, 84%, 65%, and 80% were found in the CIA+melatonin, compared to control treatment.

### 3.2. Growth, Yield, Photosynthetic Pigments, and Moisture Content of Spinach

The data regarding plant height, Shoots DW, and Roots DW were 12.1–31.2 cm, 1.02–2.02, and 0.56–1.39 g pot^−1^, respectively (Table 4). Interestingly, compared to the control, the greatest improvements were achieved by 157%, 98%, and 148% in plant height, Shoots DW, and Roots DW, respectively, with CIA+melatonin treatment. The CLR–a and CLR–b contents were in the ranges of 0.98–1.92 and 0.67–1.59 mg g^−1^ FW, whereas RTW values were from 83.3–96.4% (Table 4). The greatest improvements of 16%, 96%, and 137% in RTW values and the contents of CLR–a and CLR–b, respectively, were found with CIA+melatonin treatment, compared to the plants grown in control.

### 3.3. Leaf Dietary Value

**Figure 2:**
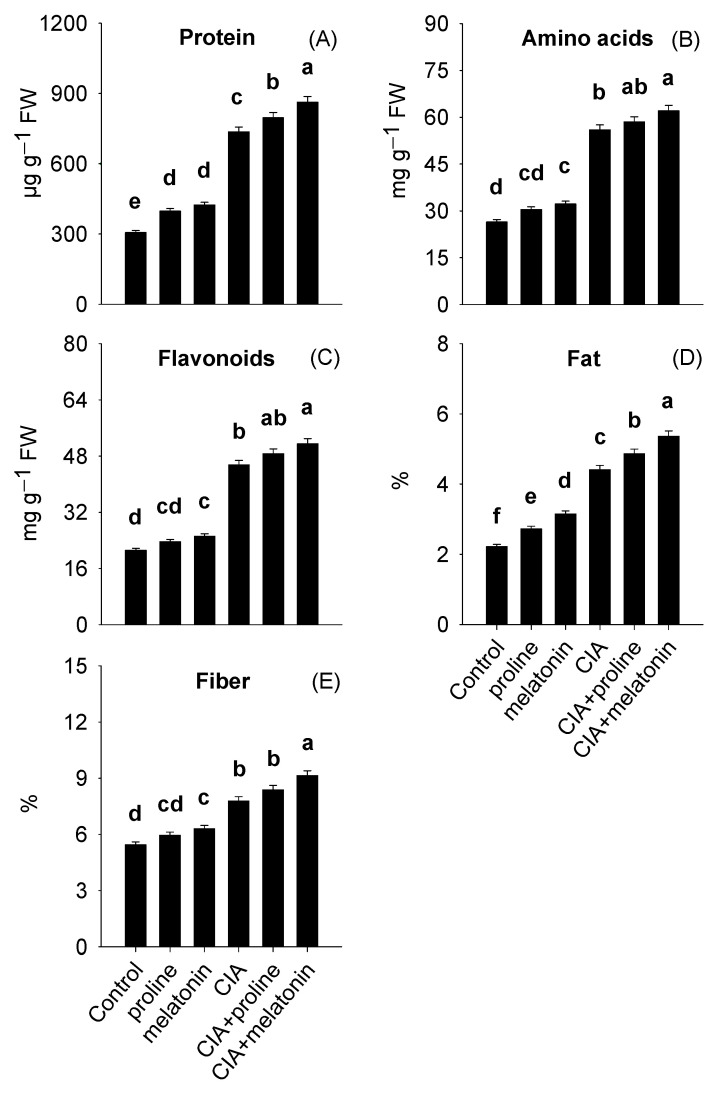
Protein (**A**), amino acids (**B**), flavonoids (**C**), fat (**D**), and fiber contents (**E**) in spinach reached by conditioning Pb-polluted soil with CIA and foliar application of proline and melatonin, as a single treatment or combining both techniques. Data shown in each bar are mean ± SE (*n* = 3). Significant variations (*p* < 0.05) based on the LSD test are shown by bars with different lowercase letters.

Protein, amino acids, flavonoids, fat, and fiber contents ranged from 306.3–863.0 µg g^−1^ FW, 26.5–62.1, 21.2–51.5 mg g^−1^ FW, 2.22–5.36, and 5.45–9.14%, respectively (Figure 2A–E). The CIA+melatonin treatment showed the greatest improvements in leaf protein, fat, fiber, amino acids, and flavonoids by 182%, 142%, 68%, 134%, and 143%, respectively, compared to the plants of control.

**Figure 3:**
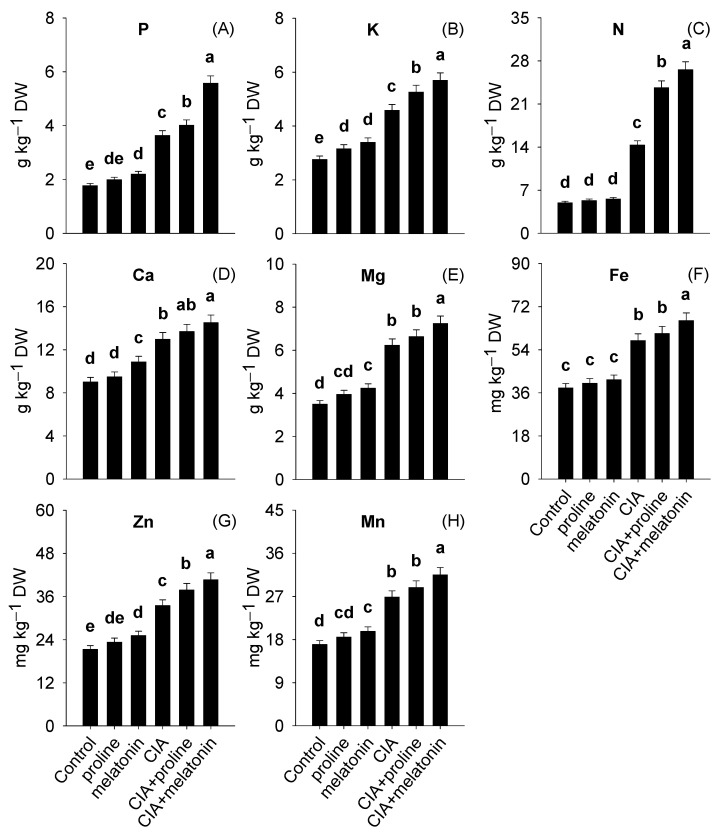
Concentrations of P (**A**), K (**B**), N (**C**), Ca (**D**), Mg (**E**), Fe (**F**), Zn (**G**), and Mn (**H**) in the leaves of spinach as affected by conditioning Pb-polluted soil with CIA and foliar application of proline and melatonin, as a single treatment or combining both techniques. Data shown in each bar are mean ± SE (*n* = 3). Significant variations (*p* < 0.05) based on the LSD test are shown by bars with different lowercase letters.

Phosphorus, K, N, Ca, and Mg concentrations in the leaves ranged from 1.77 to 5.58, 2.75–5.70, 4.96–23.7, 9.00–14.5, and 3.49–7.24 g kg^−1^ DW, while Fe, Zn, and Mn concentrations ranged from 38.1–66.2, 21.3–40.7, and 17.0–31.5 mg kg^−1^ DW (Figure 3A–H). Interestingly, topmost improvements in P, K, N, Mg, Fe, Zn, Mn, and Ca concentrations were achieved with CIA+melatonin treatment by 215%, 107%, 436%, 107%, 74%, 91%, 86%, and 61% respectively, in contrast with the plants of untreated pots.

### 3.4. Stress Response and Antioxidant Defense System

As shown in Table 3, activities of APX, CAT, and SOD, contents of AsA, and DHAR, were 0.39–1.27 and 25.7–93.7 µmol min^−1^ protein, 42.9–157.0 U min^−1^ mg^−1^ protein, 559.3–1474.3 nmol g^−1^ FW, and 26.5–81.9 µmol min^−1^ protein, respectively. Whereas contents of H_2_O_2_ and MDA, and O_2_^•−^ generation rate in leaves, were in the ranges of 21.2–66.9 and 15.9–60.3 nmol g^−1^ FW, and 12.7–39.6 nmol min^−1^ g^−1^ FW, respectively (Table 3). The CIA+melatonin treatment showed the highest significant improvements by 226%, 264%, 266%, 209%, and 164%, in APX, CAT, and SOD activities and DHAR and AsA contents, compared to the plants in untreated pots. Moreover, contents of H_2_O_2_, MDA, and O_2_^•−^ in leaves were significantly lowered by 68%, 68%, and 74%, compared to the control.

### 3.5. Enzymes, Microbial Numbers, and MBC in the Soil

**Figure 4:**
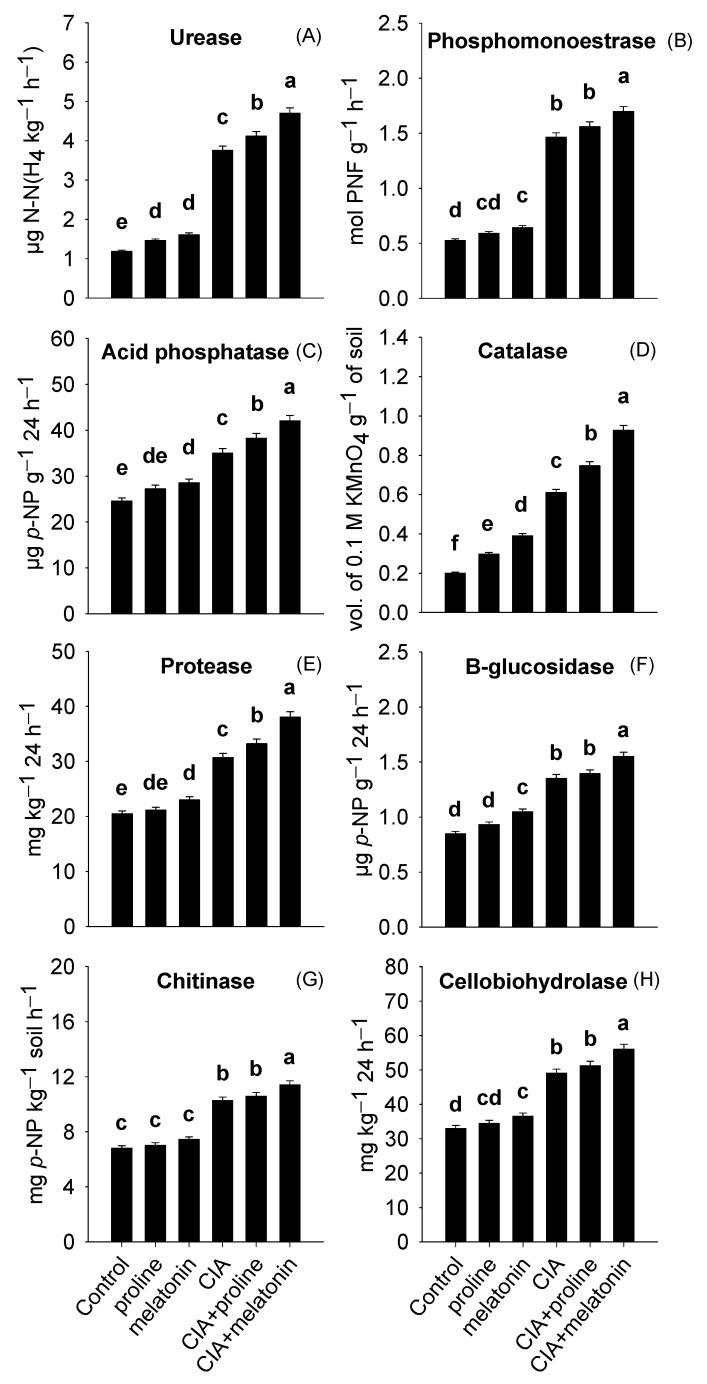
Activities of urease (**A**), phosphomonoestrase (**B**), acid phosphatase (**C**), catalase (**D**), protease (**E**), β-glucosidase (**F**), chitinase (**G**), and cellobiohydrolase (**H**) in the soil as influenced by conditioning Pb-polluted soil with CIA and foliar application of proline and melatonin, as a single treatment or combining both techniques. Data shown in each bar are mean ± SE (*n* = 3). Significant variations (*p* < 0.05) based on the LSD test are shown by bars with different lowercase letters.

Activities of urease, phosphomonestrase, acid phosphatase, and catalase were in the ranges of 1.19–4.70 µg N–N(H_4_ kg^−1^ h^−1^), 0.53–1.70 mol PNF g^−1^ h^−1^, 24.6–42.1 µg *p*–NP g^−1^ 24 h^−1^, and 0.20–0.93 Vol. of 0.1 M KMnO_4_ g^−1^ of soil (Figure 4A–D). Likewise, protease, β-glucosidase, chitinase, and cellobiohydrolase activities varied from 20.5–38.0 mg kg^−1^ 24 h^−1^, 0.85–1.55 µg *p*–NP g^−1^ 24 h^−1^, 6.81 to 11.4 mg *p*–NP kg^−1^ soil h^−1^, and 33.0–56.0 mg kg^−1^ 24 h^−1^, respectively (Figure 4E–H). The CIA+melatonin treatment showed significant improvements by 86%, 82%, 70%, 68%, 295%, 220%, 71%, and 363% in protease, β-glucosidase, cellobiohydrolase, chitinase, urease, phosphomonestrase, acid phosphatase, and catalase activities in the soil, compared to the soil of control treatment.

**Figure 5:**
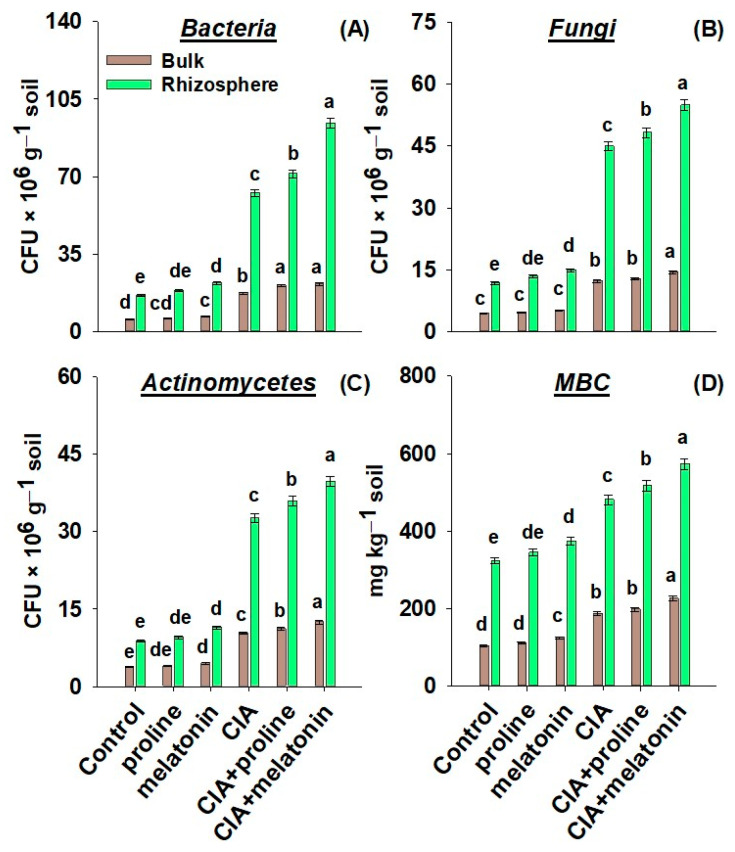
The numbers of bacteria (**A**), fungi (**B**), and actinomycetes (**C**), as well as microbial biomass carbon (MBC) (**D**), in the bulk and rhizosphere portions of the soil as affected by amending Pb-polluted soil with CIA and foliar application of proline and melatonin, as a single treatment or combining both techniques. Data shown in each bar are mean ± SE (*n* = 3). Significant variations (*p* < 0.05) based on the LSD test are shown by bars with different lowercase letters.

The numbers of bacteria, fungi, and actinomycetes were in the ranges of 5.66–21.5, 4.60–14.5, and 3.77–12.5 CFU × 10^6^ g^−1^ soil in the bulk soil portion, while from 16.3–94.2, 11.9–54.9, and 8.8–39.7 CFU × 10^6^ g^−1^ soil in rhizosphere soil portion (Figure 5A–C). The MBC contents were in the ranges of 103.7–226.2 mg kg^−1^ soil in the bulk soil portion, while from 323.6–572.9 mg kg^−1^ soil in the rhizosphere soil portion (Figure 5D). With each treatment, MBC contents and the numbers of bacteria, actinomycetes, and fungi were higher in the rhizosphere soil than in bulk soil. The highest improvements by 361%, 476%, 351%, and 77% in the numbers of fungi, bacteria, and actinomycetes, as well as MBC content in the rhizosphere soil and till of 214%, 279%, 230%, and 218% in the bulk soil, correspondingly, were found with CIA+melatonin treatment, compared to their matching controls.

## 4. Discussion

### 4.1. Lead Concentrations in Plant Parts and Plant-Accessible Pb

Soil pollution with Pb resulted in high values of ST(Pb), RT(Pb), BAF(ST), and BAF(RT) in spinach [6] and cotton [61]. The most important data related to the reduced PbDTPA and PbH_2_O in soil (Figure 1F,G), the values of ST(Pb) and RT(Pb) (Figure 1A,B), as well as their BAF(ST), BAF(RT), and TF(ST) values (Figure 1C–E), were achieved with CIA+melatonin treatment. Formerly, amending the Pb-polluted soil with BH and MKC reduced Pb uptake in spinach shoots [6]. Similarly, Vannini et al. [62] confirmed that the addition of BH (5% *w*/*w*) in Pb-contaminated soil effectively reduced soil bioavailable fraction of Pb (up to 50%) and its accumulation in lettuce shoots (up to 50%). The lower availability of Pb in the soil and its uptake by spinach is because of multiple mechanisms associated with BH and MKC in the CIA, which are as follows: (i) the hydration process of MKC within the soil contributed to the conversion of Pb into extremely insoluble Pb-based compounds and struvite-K encapsulation [63] and (ii) the presence of extensive functional groups (like hydroxyl, carboxyl, and phenolic) on BH surfaces efficiently adsorbed Pb^2+^ through precipitation, electrostatic interaction, and surface complexation [13]. Apart from it, the decline in Pb concentrations in spinach is also due to the formation of PCs under the effect of melatonin. These PCs formed Pb–PC complexes which were later sequestered and compartmentalized in the vacuoles of roots and reduced Pb movement to aerial portions [17].

### 4.2. Growth, Yield, Photosynthetic Pigments, and Moisture Content of Spinach

High concentrations of Pb in soil reduced the photosynthetic activities and RTW in soybean [64] and wheat [65]. Additionally, growth retardation and limited biomass of *Athyrium wardii* [66] and cotton [61] raised on Pb-contaminated soils were observed. In our experiment, compared to untreated pots, the greatest improvement in RTW values, contents of photosynthetic pigments, growth, and yield were achieved in the CIA+melatonin treatment (Table 4). Adding BH in Pb-contaminated soil improved RTW values, concentrations of CLR–a and CLR–b, and shoots DW of spinach [13]. In another experiment, the lengths of roots and shoots of black-eyed peas were significantly increased with 25% of _n_Si (silica nanoparticles from coir pith) compared to other _n_Si concentrations [67]. Our data correlate with the findings of [6], where the beneficial roles of BH and MKC on biophysical characteristics of spinach under Pb stress were reported. Furthermore, spraying melatonin improved the growth and yield of maize [21] and safflower [17] grown in Pb-stressed soils. The improvements in RTW values, photosynthetic pigments, and growth of spinach are due to several mechanisms linked with the CIA, which are as follows: (1) BH upgraded soil health through increasing moisture retention, porosity, CEC, plant–water relations, and supply of nutrients [13] and (2) CIA mediated reduction in bioavailable Pb in the soil reduced Pb toxicity to spinach [15]. Melatonin also improved the ability of spinach to uptake nutrients from the soil via improving the architecture of the root system [68]. Melatonin protected chloroplast from damage and helped to synthesize chlorophyll by regulating nitrous oxide (NO) [69].

### 4.3. Leaf Dietary Value

Exposure to Pb stress reduces the uptake of nutrients through plant roots, thus negatively affecting the production of biochemical compounds [1]. The CIA+melatonin treatment showed the most significant data regarding the highest increment in the contents of protein, amino acids, flavonoids, fat, and fiber (Figure 2A–E), and nutrient concentrations in leaves (Figure 3A–H). Formerly, BH and MKC addition to Pb-polluted soils improved nutrients and biochemical compounds in spinach and grasses [6,70]. Furthermore, the improvements in nutrition and biochemical compounds of maize [21] and tomato [71] were also reported with foliar application of melatonin. Biochar improved spinach traits by improving plant–water relations [10] providing nutrients to the plants [20] and enhancing the biogeochemical cycling of nutrients by improving the activities of microorganisms in the soil [6]. Moreover, melatonin helped to improve the dietary value of spinach by raising the activity of H^+^–ATPase, which enhances the ability of a plant to uptake nutrients [69]. This uptake of nutrients also led to improving the biochemical status of spinach by enhancing various metabolic processes [71]. Higher flavonoids, anthocyanins, and protein contents in spinach are due to melatonin-mediated improvement in soluble sugar concentrations, which the plants later consume to synthesize these compounds [72,73,74].

### 4.4. Stress Response and Antioxidant Defense System

Soil pollution with Pb increases the production of MDA and H_2_O_2_ in plants, suppressing the activities of antioxidant enzymes [75]. In our study, the highest activities of antioxidants and least ROS constituents were observed in plants of CIA+melatonin treatment, compared to untreated pots (Table 3). Previously, amending Pb-polluted soil with MKC improved antioxidant enzymes in spinach [6]. Furthermore, significant reductions in the contents of ROS were observed in spinach when grown on BH-amended Pb-polluted soil [10]. Improvements in the activities of antioxidants while reducing oxidative stress in maize [21] and safflower [17] were reported with the foliar application of melatonin. In our case, the entry of Pb was efficiently reduced in spinach plants because Pb was immobilized in soil with BH and MKC present in the CIA amendment. This decline in Pb concentrations resulted in lower production of ROS, thus reducing lipid peroxidation in plants [6,13]. In addition, melatonin spray on spinach prevented lipid peroxidation, initially through the formation of Pb–PC complexes and later, their sequestration and compartmentalization in the vacuoles of roots and leaves [17]. Melatonin reduced lipid peroxidation in spinach by reducing H_2_O_2_ toxicity to the plants and degrading H_2_O_2_. Melatonin boosted the synthesis of anthocyanins and carotenoids in spinach plants, which scavenged MDA and H_2_O_2_, reducing lipid peroxidation [76].

### 4.5. Enzymatic Activities, Microbial Numbers, and MBC in the Soil

Soil pollution with Pb negatively influences the activities of urease, catalase, acid phosphatase, phosphomonoestrase, protease, and β-glucosidase in Pb-contaminated soils because Pb directly alters the numbers and diversity of soil microbiota [61,66]. In the current study, the CIA+melatonin treatment reflected the maximum numbers of bacteria, fungi, and actinomycetes, and the values of MBC in bulk and rhizosphere soil sections (Figure 5A–D) along with enzymes, compared to control (Figure 4A–H). Our findings are endorsed by the previous investigations where increments in MBC, activities of soil enzymes, and microbial numbers in metal-contaminated soils were found after the addition of BH [77,78].

Several mechanisms driven by BH and MKC improved MBC, microbial numbers, and soil enzyme activities in the soil. Upon the immobilization of Pb with BH [15] and MKC [63], Pb toxicity was reduced to microbial communities, which enhanced not only their masses but also various enzymes secreted by them in the soil [6]. Moreover, BH also reshaped the microbial populations in the soil by improving porosity, moisture retention, and carbon levels (Liu et al., 2018). Melatonin also contributed to enhancing microbial-associated parameters by improving the growth of spinach roots, thus offering more space for microorganisms in the rhizosphere [68,79]. With melatonin application, root exudate secretion, particularly malate and citrate, is enhanced in the rhizosphere [80]. These exudates supported bacterial and fungal communities in the spinach rhizosphere, which released peroxidase, dehydrogenase, and other enzymes [81,82].

## 5. Conclusions

The experimental results conclude that exogenous application of melatonin to spinach plants resulted in greater reductions of ST(Pb), RT(Pb), and PbH_2_O in soil, as well as increased levels of photosynthetic pigments, plant growth, antioxidant enzymes, biochemical compounds, nutrients, soil enzymatic activities, and microbiota. Similarly, the effects of both treatments, CIA and CIA+proline, on reducing the ST(Pb), RT(Pb), and PbH_2_O in soil, ROS, and enhancing the majority of nutrients and soil enzyme activities were comparable. However, conditioning Pb-contaminated soil with CIA and foliar melatonin spray (CIA+melatonin) has the greatest effect on reducing ST(Pb), RT(Pb), PbH_2_O in soil, and PbDTPA, as well as oxidative stress in plants. Additionally, CIA+melatonin treatment resulted in the greatest increases in microbial numbers (both in bulk and rhizosphere soil portions), activities of soil enzymes and plant antioxidant enzymes, concentrations of nutrients, and biochemical compounds in leaves. The results confirmed that conditioning Pb-contaminated soil with CIA and foliar melatonin spray (CIA+melatonin) is a low-cost and innovative strategy for reducing the mobility of Pb in soil and its uptake by spinach. However, several crucial points such as the dose of CIA, the concentration of melatonin spray, Pb concentrations in the soil, crop type, and soil physicochemical properties should be kept in mind before using this technique at the field level where food safety is a vital matter.

## Figures and Tables

**Table 1:** Physicochemical attributes of soil are as follows. The data herewith are the mean of three replicates ± standard deviation.

Characteristics	Units	Values
Soil texture	-	Clay loam
Clay	g kg^−1^	301 ± 3.51
Silt	g kg^−1^	286 ± 2.90
Sand	g kg^−1^	413 ± 4.82
pH (H_2_O)	−	7.10 ± 0.02
EC ^a^	dSm^−1^	3.7 ± 0.03
CEC ^b^	cmol_c_ kg^−1^	24.9 ± 0.52
OM ^c^	g kg^−1^	7.11 ± 0.04
CaCO_3_	g kg^−1^	27.8 ± 0.82
DTPA–extractable Pb	mg kg^−1^	0.89 ± 0.07
Total Pb	mg kg^−1^	21.24 ± 1.06
Exchangeable K	mg kg^−1^	87.0 ± 2.97
Available P	mg kg^−1^	91.0 ± 3.21

^a^ Electrical conductivity. ^b^ Cation exchange capacity. ^c^ Organic matter.

**Table 2:** Treatments used in the study.

Treatments	Acronyms	The Dose of CIA Added to the Soil	Concentrations of Foliar Spray	
		(%)	PL (mM L^−1^)	ML (µM L^−1^)
Control	Control	-	-	-
Proline	Proline	-	20	-
Melatonin	Melatonin	-	-	100
Combo immobilizing amendment	CIA	3	-	-
Combo immobilizing amendment+proline	CIA+proline	3	20	-
Combo immobilizing amendment+melatonin	CIA+melatonin	3	-	100

**Table 3:** Responses of antioxidants and ROS in spinach grown on Pb-contaminated soil as affected by amending Pb-polluted soil with CIA and foliar application of proline and melatonin, as a single treatment or combining both techniques. Data are the mean of three replicates (±standard error, SE). Different letters indicate a significant variation at *p* < 0.05 among the treatments.

Treatments	APX	SOD	CAT	AsA	DHAR	H_2_O_2_	O_2_^•−^	MDA
	(µmol min^−1^ Protein)	(U min^−1^ mg^−1^ Protein)	(µmol min^−1^ Protein)	(nmol g^−1^ FW)	(µmol min^−1^ Protein)	(nmol g^−1^ FW)	(nmol min^−1^ g^−1^ FW)	(nmol g^−1^ FW)
Control	0.39 ± 0.11 ^e^	42.9 ± 1.22 ^d^	25.7 ± 0.73 ^e^	559.3 ± 15.6 ^d^	26.5 ± 0.75 ^d^	66.9 ± 1.89 ^a^	39.6 ± 1.10 ^a^	60.3 ± 1.71 ^a^
Proline	0.46 ± 0.01 ^d^	50.5 ± 1.43 ^cd^	30.5 ± 0.87 ^de^	714.3 ± 20.0 ^c^	32.4 ± 0.90 ^c^	58.1 ± 1.63 ^b^	35.7 ± 0.99 ^b^	52.8 ± 1.48 ^b^
Melatonin	0.52 ± 0.01 ^d^	56.6 ± 1.59 ^c^	33.1 ± 0.93 ^d^	778.7 ± 21.8 ^c^	37.0 ± 1.04 ^c^	53.3 ± 1.48 ^c^	31.7 ± 0.87 ^c^	48.1 ± 1.36 ^c^
CIA	0.96 ± 0.03 ^c^	142.3 ± 4.00 ^b^	78.6 ± 2.21 ^c^	1297.3 ± 36.5 ^b^	69.4 ± 1.94 ^b^	27.8 ± 0.78 ^d^	17.1 ± 0.46 ^d^	22.0 ± 0.61 ^d^
CIA+proline	1.15 ± 0.03 ^b^	148.6 ± 4.18 ^ab^	85.6 ± 2.41 ^b^	1386.7 ± 38.9 ^ab^	74.1 ± 2.09 ^b^	25.2 ± 0.70 ^d^	15.7 ± 0.43 ^d^	18.7 ± 0.52 ^de^
CIA+melatonin	1.27 ± 0.03 ^a^	157.0 ± 4.41 ^a^	93.7 ± 2.64 ^a^	1474.3 ± 41.5 ^a^	81.9 ± 2.29 ^a^	21.2 ± 0.58 ^e^	12.7 ± 0.35 ^e^	15.9 ± 0.44 ^e^
References of the protocols	[47]	[48]	[49]	[50,51]	[47]	[52]	[53]	[54]

APX = ascorbate peroxidase; SOD = superoxide dismutase; CAT = catalase; AsA = ascorbic acid; DHAR = dehydroascorbate reductase; H_2_O_2_ = hydrogen peroxide; O_2_^•−^ = superoxide anion, and MDA = Malondialdehyde.

**Table 4:** Plant height, biomass, pigments, and relative water content of spinach planted on Pb-contaminated soil as affected by conditioning Pb-polluted soil with CIA and foliar application of proline and melatonin, as a single treatment or combining both techniques. Data are the mean of three replicates (±standard error, SE). Different letters indicate a significant difference at *p* < 0.05 among the treatments.

Treatments	Plant Height (cm)	Shoots DW (g pot^−1^)	Roots DW (g pot^−1^)	CLR–a (mg g^−1^ FW)	CLR–b (mg g^−1^ FW)	RTW (%)
Control	12.1 ± 0.35 ^e^	1.02 ± 0.03 ^e^	0.56 ± 0.01 ^e^	0.98 ± 0.03 ^e^	0.67 ± 0.02 ^d^	83.3 ± 1.19 ^b^
Proline	13.6 ± 0.38 ^de^	1.19 ± 0.03 ^d^	0.65 ± 0.02 ^d^	1.20 ± 0.03 ^d^	0.79 ± 0.02 ^c^	84.9 ± 1.19 ^b^
Melatonin	15.4 ± 0.44 ^d^	1.29 ± 0.03 ^d^	0.80 ± 0.02 ^c^	1.33 ± 0.04 ^d^	0.89 ± 0.03 ^c^	86.1 ± 1.22 ^b^
CIA	26.5 ± 0.75 ^c^	1.72 ± 0.05 ^c^	1.23 ± 0.03 ^b^	1.57 ± 0.04 ^c^	1.38 ± 0.04 ^b^	94.5 ± 1.33 ^a^
CIA+proline	28.6 ± 0.78 ^b^	1.85 ± 0.05 ^b^	1.31 ± 0.04 ^ab^	1.76 ± 0.05 ^b^	1.47 ± 0.04 ^b^	95.8 ± 1.37 ^a^
CIA+melatonin	31.2 ± 0.87 ^a^	2.02 ± 0.06 ^a^	1.39 ± 0.04 ^a^	1.92 ± 0.05 ^a^	1.59 ± 0.04 ^a^	96.4 ± 1.37 ^a^
LSD_0.05_ value	1.95	0.13	0.09	0.13	0.10	3.94

Shoots DW = shoots dry weight; Roots DW = roots dry weight; CLR–a = Chlorophyll–a; CLR–b = Chlorophyll–b, and RTW = relative water content.

## Data Availability

Data generated or analyzed during this study are included in this article.
